# Metabolic profiles of triple-negative and luminal A breast cancer subtypes in African-American identify key metabolic differences

**DOI:** 10.18632/oncotarget.24433

**Published:** 2018-02-07

**Authors:** Fariba Tayyari, G.A. Nagana Gowda, Olufunmilayo F. Olopade, Richard Berg, Howard H. Yang, Maxwell P. Lee, Wilfred F. Ngwa, Suresh K. Mittal, Daniel Raftery, Sulma I. Mohammed

**Affiliations:** ^1^ Complex Carbohydrate Research Center, University of Georgia, Athens, Georgia 30602, USA; ^2^ Northwest Metabolomics Research Center, Department of Anesthesiology and Pain Medicine, University of Washington School of Medicine, Seattle, WA 98109, USA; ^3^ The University of Chicago Medical Center, Chicago, IL 60637, USA; ^4^ Indiana University Health Arnett Medical, Lafayette, IN 47905, USA; ^5^ Laboratory of Cancer Biology and Genetics, Center for Cancer Research, National Cancer Institute, NIH, Bethesda, MD 20892, USA; ^6^ Brigham and Women’s Hospital, Dana-Farber Cancer Institute, and Harvard Medical School, Boston, MA 02115, USA; ^7^ Depatment of Comparative Pathobiology and Purdue University Center for Cancer Research, Purdue University, West Lafayette, IN 47907, USA; ^8^ Division of Public Health Sciences, Fred Hutchinson Cancer Research Center, Seattle, WA 98109, USA

**Keywords:** breast cancer, triple-negative, NMR, African American, Caucasian

## Abstract

Breast cancer, a heterogeneous disease with variable pathophysiology and biology, is classified into four major subtypes. While hormonal- and antibody-targeted therapies are effective in the patients with luminal and HER-2 subtypes, the patients with triple-negative breast cancer (TNBC) subtype do not benefit from these therapies. The incidence rates of TNBC subtype are higher in African-American women, and the evidence indicates that these women have worse prognosis compared to women of European descent. The reasons for this disparity remain unclear but are often attributed to TNBC biology. In this study, we performed metabolic analysis of breast tissues to identify how TNBC differs from luminal A breast cancer (LABC) subtypes within the African-American and Caucasian breast cancer patients, respectively. We used High-Resolution Magic Angle Spinning (HR-MAS) 1H Nuclear magnetic resonance (NMR) to perform the metabolomic analysis of breast cancer and adjacent normal tissues (total n=82 samples). TNBC and LABC subtypes in African American women exhibited different metabolic profiles. Metabolic profiles of these subtypes were also distinct from those revealed in Caucasian women. TNBC in African-American women expressed higher levels of glutathione, choline, and glutamine as well as profound metabolic alterations characterized by decreased mitochondrial respiration and increased glycolysis concomitant with decreased levels of ATP. TNBC in Caucasian women was associated with increased pyrimidine synthesis. These metabolic alterations could potentially be exploited as novel treatment targets for TNBC.

## INTRODUCTION

Breast cancer is a heterogeneous group of diseases that are immunohistochemically categorized by the cancer cell expression of the estrogen receptor (ER), progesterone receptor (PR), and human epidermal growth factor receptor-2 (HER-2) amplification into four main subtypes: luminal A, luminal B, Her-2-positive, and triple-negative [1][2]. These subtypes not only differ in their hormonal statuses and HER-2 expressions, but also clinically vary in their prognoses, responses to therapy, and incidence rates [3]. The incidence rates of TNBC are higher in younger premenopausal women and African-American women than in Caucasian women [4].

TNBC constitutes about 10-20% of all diagnosed breast cancer cases. Despite its smaller percentage, TNBC causes a disproportionate number of breast cancer deaths due to its aggressive nature, earlier relapses, and distinct patterns of metastasis [5]. Because of its lack of hormonal receptors (ER and PR) and HER-2 receptor, TNBC is not responsive to hormone or anti-HER-2 monoclonal antibody therapy. Current TNBC treatment is limited to systemic cytotoxic chemotherapy [6]. On the other hand, LABC typically constitutes 40-50% of invasive breast cancer, is more prevalent in Caucasian women, and has a good prognosis.

In the pursuit of identifying specific targeted therapy for TNBC, recent preclinical studies have identified potential molecular targets, including the epidermal growth factor receptor (EGFR), SRC, MET, and poly ADP ribose polymerase 1/2 (PARP1/2). However, these targets have underperformed in their clinical testing [5]. Therefore, the need to identify an effective treatment for TNBC still exists. This could be achieved by a better understanding of its biology, which may aid in the discovery of new specific therapeutic targets. Many approaches including proteomic and genomic techniques have been utilized to study the biology of TNBC [7] [8] [9] and more recently metabolomics methods have been used as a promising alternative approach [10]. Because metabolites are sensitive to subtle differences in an individual’s pathological status, their profiling may identify altered pathways and key enzymes that could provide novel therapy targets. A variety of powerful analytical techniques such as nuclear magnetic resonance (NMR) and mass spectrometry (MS), and multivariate statistical methods [11, 12] have been shown to reliably identify differentially altered metabolites in various biological samples [13, 14].

In this study, we used a metabolomic approach to identify the altered metabolites of TNBC (ER, PR, HER-2 negative subtype) and LABC (ER and PR positive and HER-2-negative subtype) within (African American women) and across race (in comparison to Caucasian women). Cancer and normal adjacent tissue samples obtained from African-American and Caucasian patients before neoadjuvant chemotherapy were studied using 1H HR-MAS NMR and multivariate statistics methods. The analysis of these tissues indicated distinct metabolic profiles and pathways of TNBC and LABC in both group of women.

## RESULTS

### Patient characteristics

The demographic and hormonal characteristics of African-American and Caucasian women’s breast cancer tissue samples (n=47, 30 African Americans, 17 Caucasians) and normal adjacent tissues (n=35, 18 African Americans, 17 Caucasians) are summarized in Table 1.

**Table 1:** Clinicopathological characteristics of women with invasive breast cancer

Patient Characteristics	Number
Total number of patients	82
African American women	48 (T=30; N=18)
Caucasian women	34 (T=17; N=17)
**Pathology**	
Invasive carcinoma grade I and DCIS	3
Invasive carcinoma grade II	10
Invasive carcinoma grade III	25
Unknown	9
**Patient Age (years)**	
**All patients**	
<50	10
>50	37
**African American women**	
<50	7
>50	23
**Caucasian women**	
<50	3
>50	14
**ER Status**	
**All patients**	
TNBC	18
LABC	29
**African American women**	
TNBC	13
LABC	17
**Caucasian women**	
TNBC	5
LABC	12

T: tumor; N: Normal adjacent tissue, TNBC= triple-negative breast cancer (ER,PR,HER2_negative); LABC =Luminal A breast cancer (ER and PR-positive and HER-2-negative).

### Global metabolite analysis distinguishes tumor and adjacent normal tissues

**Figure 1:**
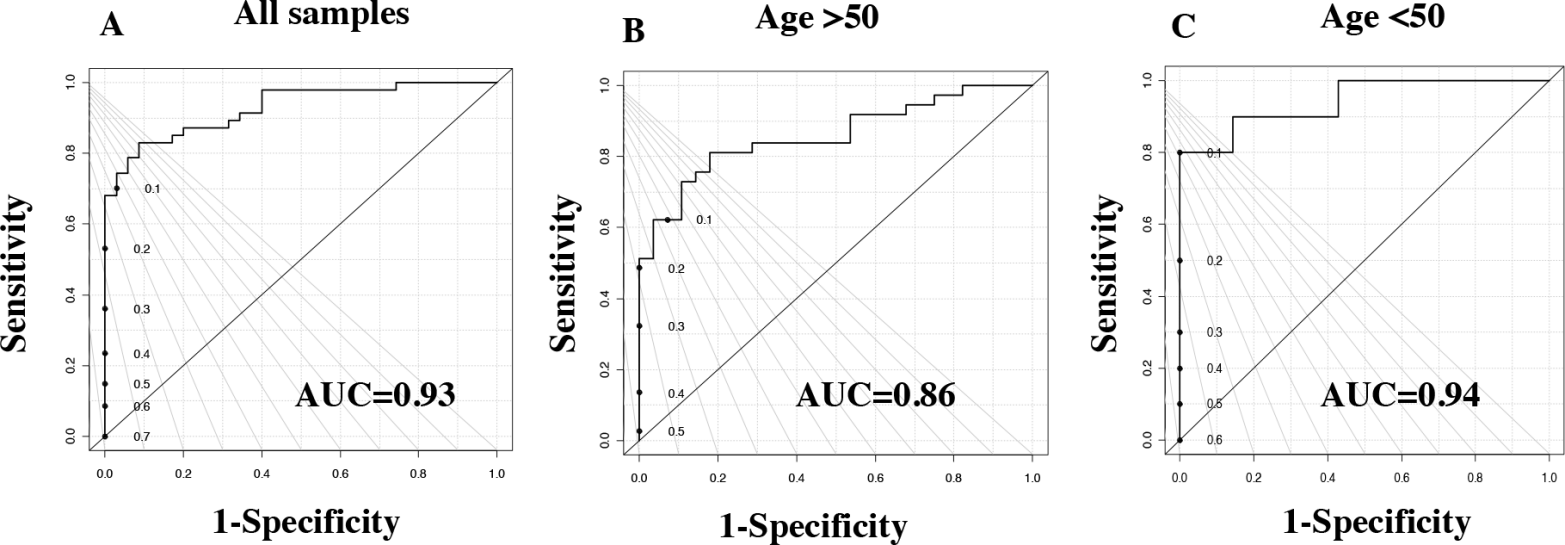
Tissue metabolite profiles derived from breast cancer patients are different from healthy control individuals ROC curve for **(A)** PLS-DA model (based on 27 measured metabolites) with 47 breast cancer patients and 35 healthy controls. **(B)** PLS-DA model (based on 27 metabolites) with postmenopausal women breast cancer tissue and adjacent control tissue with women of >50 years of age. **(C)** PLS-DA model (based on the 27 metabolites) with premenopausal women breast cancer tissue and adjacent control tissue with women of <50 years of age.

We employed HR-MAS NMR analysis to perform the metabolic profiling of TNBC and LABC in African American and Caucasian women. Qualitatively, the ^1^H NMR spectra of adjacent normal tissues were dominated by lipids signals. The tumor spectra showed the peak intensities of a large number of metabolites to be significantly higher than the normal tissues. Lipids and a number of small molecules, including amino and organic acids, were identified in the spectra and listed in Supplementary Table 1 and Supplementary Figure 1. A total of 27 metabolites were assigned to the corresponding resonances by comparing the chemical shifts and peak multiplicities to the previously reported data [15]. The normalized NMR data were analyzed by PLS-DA to differentiate the tumors from the normal adjacent tissue. The PLS-DA score plots showed a clear differentiation, indicating distinct metabolic differences between breast cancer and normal adjacent tissue samples. The ROC curve for the predictive model was derived from the PLS-DA analysis of the 27 metabolites listed in Supplementary Table 1. Figure 1A features a very good specificity of 0.9, a sensitivity of 0.8, and an AUROC of 0.93. Similar analytical results using NMR-detected metabolites from postmenopausal women (>50 years old) are presented in Figure 1B with an AUROC of 0.86. The samples from premenopausal women (<50 years old) also showed good differentiation (Figure 1C) with an AUROC of 0.94. These data suggested that patients and controls could be predicted either as breast cancer patients or healthy controls (as well as whether they were postmenopausal or pre-menopausal) with high sensitivity and specificity (Table 2).

**Table 2:** Metabolites significantly (p<0.05) expressed between all tumor and all normal tissues stratified by age

Tumor vs. Normal (All ages)		Tumor vs. Normal (> 50yrs)		Tumor vs. Normal (< 50yrs)	
Alanine	0.034	Alanine	0.004	Myo-inositol	0.040
ATP	0.006	ATP	0.012	Phosphocholine	0.049
Choline	0.012	Choline	0.001	Uridine	0.022
Creatine	0.043	Glucose	0.044		
Glutamate	0.008	Glutamate	0.001		
Glutamine	0.005	Glutamine	0.023		
Glutathione	0.003	Glutathione	0.012		
Glycine	0.032	Glycine	0.001		
Lactate	0.001	Lactate	0.001		
Myo-inositol	0.001	Lipid	0.015		
Phosphocholine	0.001	Methionine	0.004		
Taurine	0.001	Myo-inositol	0.001		
Tyrosine	0.041	Phenylalanine	0.016		
Uridine	0.034	Phosphocholine	0.005		
		Taurine	0.003		
		Threonine	0.006		
		Tyrosine	0.012		
		Unsaturated lipid	0.012		
		Valine	0.015		

Box-and-whisker plots for the peak intensities of 6 of the 27 metabolites showed that the lipids, unsaturated lipids, and ATP levels were lower in the tumors; however, the methionine, choline, and phosphocholine levels were higher in the tumors compared to the adjacent normal tissues (Supplementary Figure 2A). Box-and-whisker plots for the peak intensities of the phosphocholine, myo-inositol and uridine metabolites were significantly higher in the tumors compared to the normal adjacent tissues of premenopausal women (Supplementary Figure 2B).

### Hormonal status and race affect metabolite expression in breast cancer

**Figure 2:**
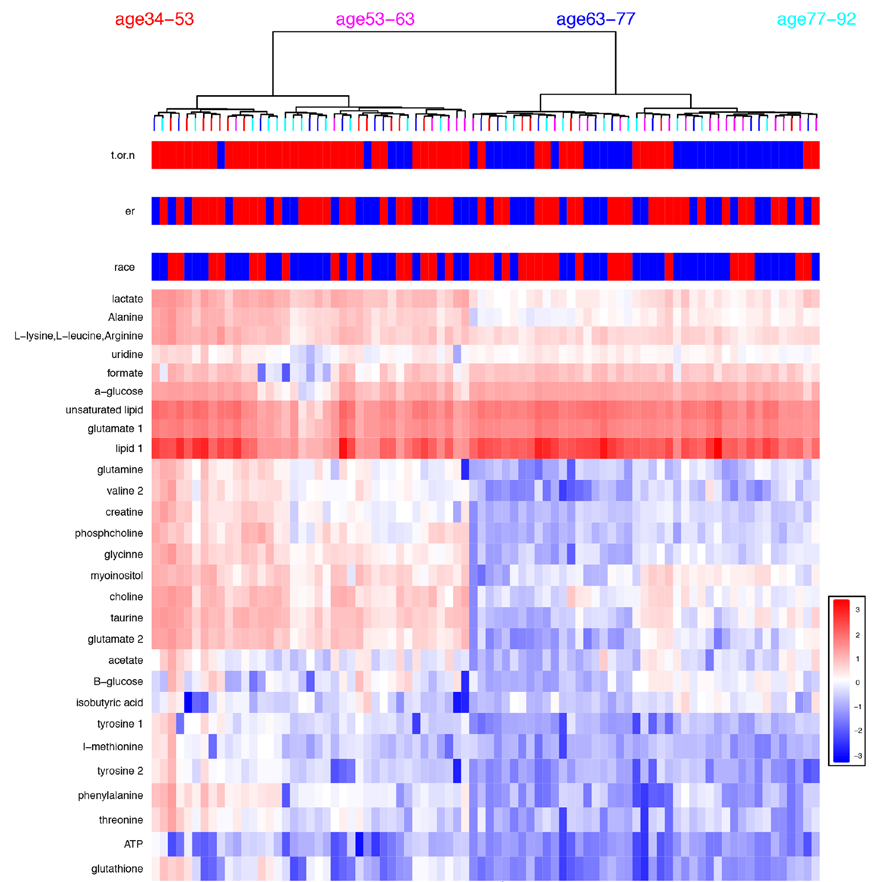

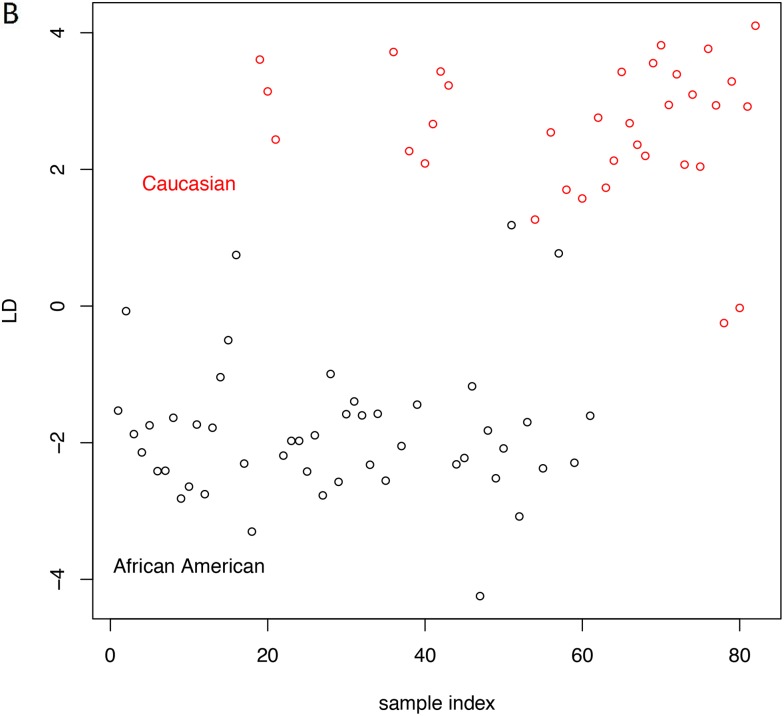

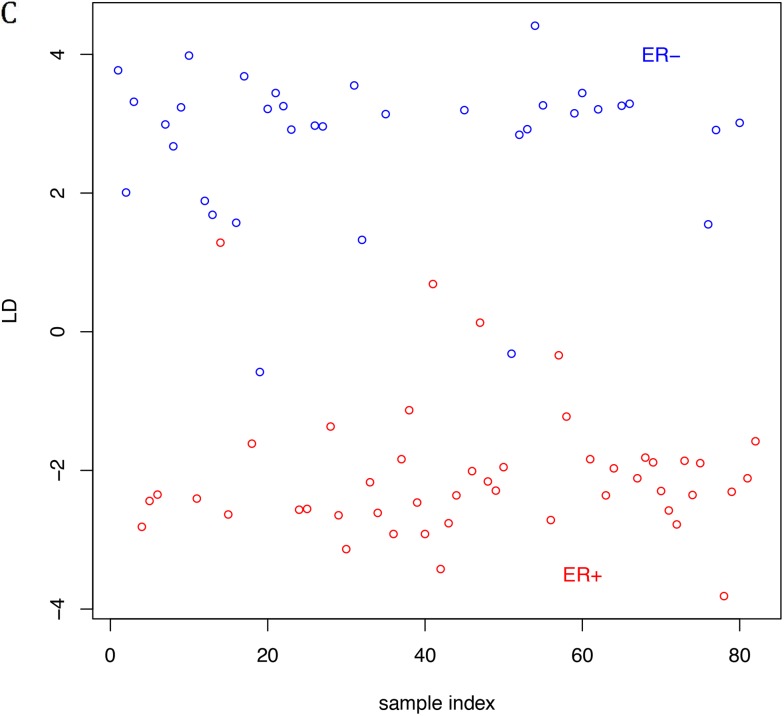
**(A)** Heatmap constructed based on clustering results from metabolite profiles of breast cancer (red) and normal (blue) samples. Heatmap features the top twenty-seven metabolites as identified by t-test analysis (p≤0.05). Distance measure is Euclidean and clustering is determined using the Ward algorithm. **(B)** Linear discriminant analysis of breast cancer samples according to race. LD plot generated for African American (black circle) and Caucasian women (red circle) breast cancer tissues. **(C)** Linear discriminant analysis of breast cancer samples according to hormonal status. LD plot generated for ER- (blue circle) and ER+ (red circle) breast cancer tissues.

We visualized the results obtained by NMR using a heatmap combined with the hierarchical clustering of tumor tissue samples by race, hormonal status, and metabolite (Figure 2). As seen in Figure 2A, the top two clusters are enriched by the tumors on the left sub-tree and the normal adjacent tissues on the right sub-tree. Using the most variable metabolites, we applied linear discriminant analysis to the tumor and normal samples and found linear discriminants that distinguish African-American from Caucasian women (Figure 2B) and ER+ from ER- (Figure 2C). Metabolites with p-values < 0.05 are listed in Table 3 for TNBC and LABC samples regardless of race.

**Table 3:** Metabolites significantly expressed (p<0.05) in luminal A breast cancer (LABC) and triple-negative breast cancer (TNBC) comparing tumors vs. adjacent normal tissue regardless of race

Metabolites	LABC *vs* their normal adjacent	TNBC *vs* their normal adjacent
Alanine	0.022	-
ATP	-	0.026
Choline	0.0046	-
Creatine	-	0.046
Glucose	-	0.020
Glutamate	0.008	-
Glutamine	0.027	-
Glutathione	0.031	0.018
Glycine	0.033	-
Lactate	0.003	0.034
Lipid	-	0.011
Methionine	0.008	-
Myo-inositol	0.007	0.013
Phosphocholine	0.006	-
Taurine	0.015	0.020
Threonine	0.012	-
Tyrosine	0.046	-
Unsaturated lipids	-	0.007
Uridine	0.016	-
Valine	0.045	-

**Figure 3:**
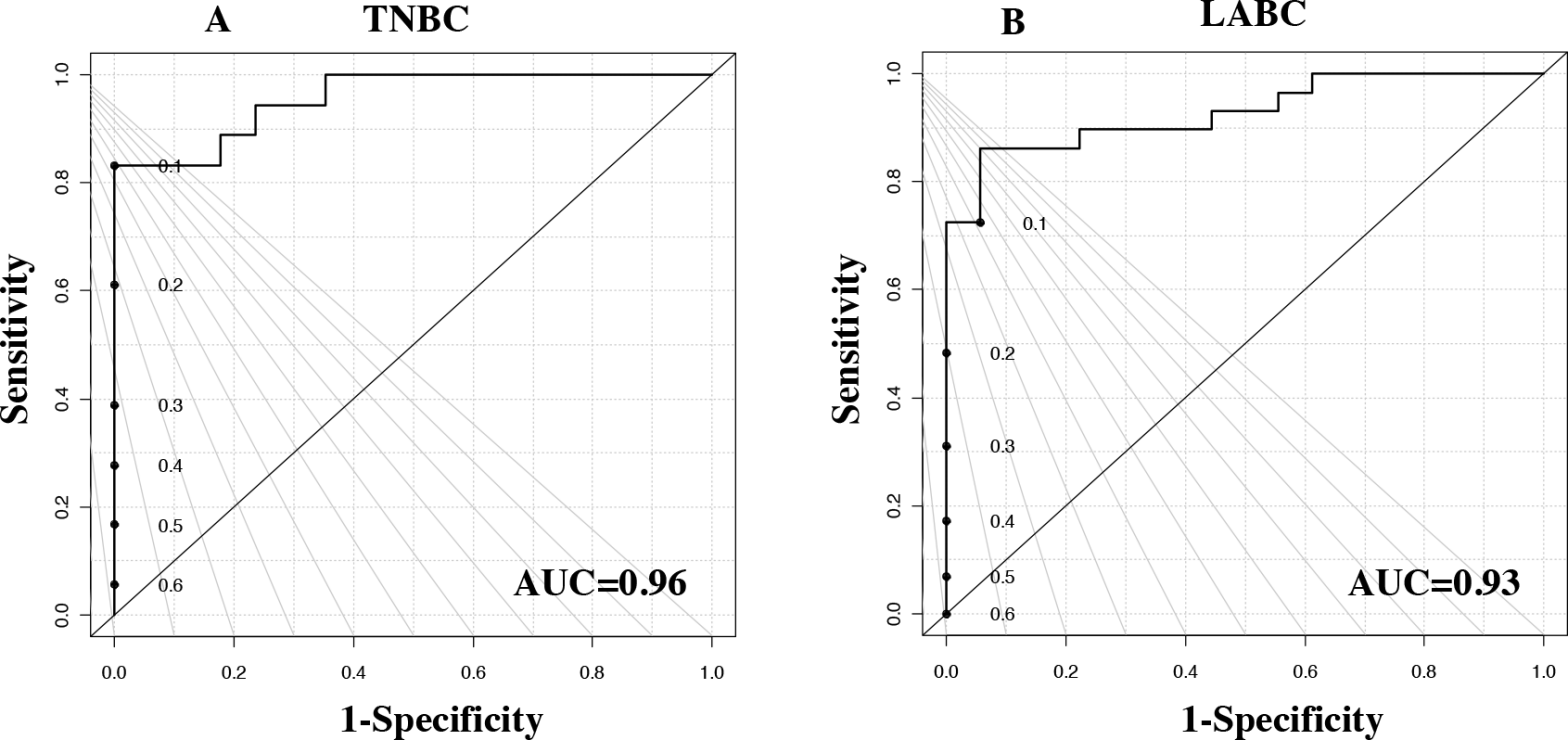
ROC curve for the results of the PLS-DA model from the 27 metabolites from **(A)** TNBC, ER-negative, samples and **(B)** LABC, ER-positive, samples.

**Figure 4:**
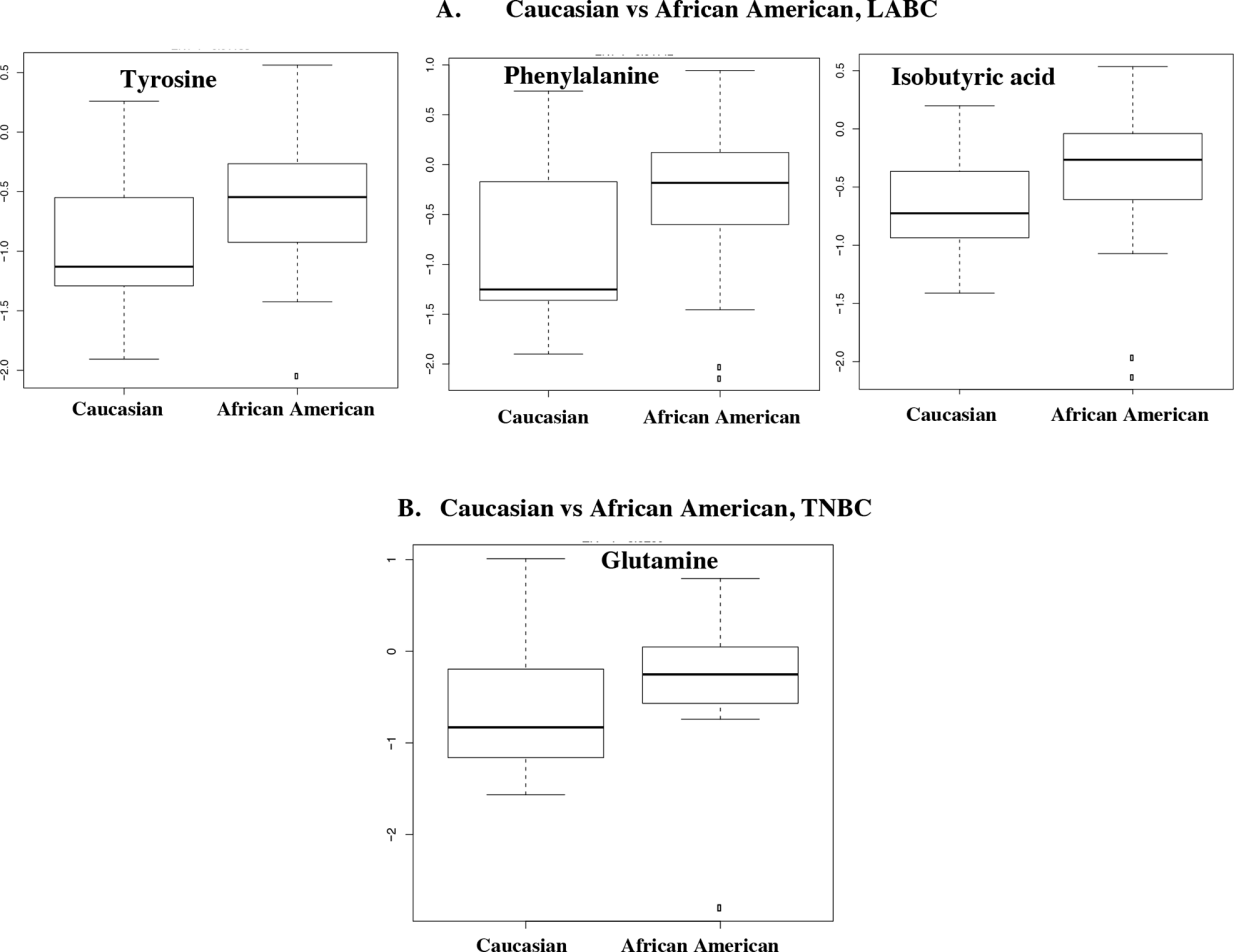
Box-and-whisker plots of metabolites with p-values < 0.05 illustrating discrimination between tumor tissues from African American and Caucasian **(A)** LABC and **(B)** TNBC. A horizontal line in the middle portion of the box represents the mean. Top and bottom boundaries of boxes show the 75th and 25th percentiles, respectively. Upper and lower whiskers show 95th and 5th percentiles, respectively. Open circles show outliers.

A PLS-DA model was developed using the 27 NMR-detected metabolites (Supplementary Table 1). We used leave-one-out cross-validation to obtain the best model and reduce over-fitting. The PLS-DA performance analyses of tumor versus adjacent normal TNBC and LABC tissue samples are featured in the ROC curves (Figures 3A and 3B). Box-and-whisker plots were created for tumor tissue metabolites with p-values <0.05 versus normal adjacent TNBC (Supplementary Figure 3) and LABC tissues (Supplementary Figure 4). With regards to the patients’ race, we found three metabolites—tyrosine, phenylalanine, and isobutyric acid—were significantly higher in the LABC of African-American women compared to the LABC of Caucasian women (Figure 4A). TNBC glutamine was the only metabolite that was significantly higher in African-American women (Figure 4B). Our data suggest that race and hormonal status affect the metabolite profiles expressed in the breast cancer patients.

### TNBC and LABC metabolite profiles of African-American women

**Figure 5:**
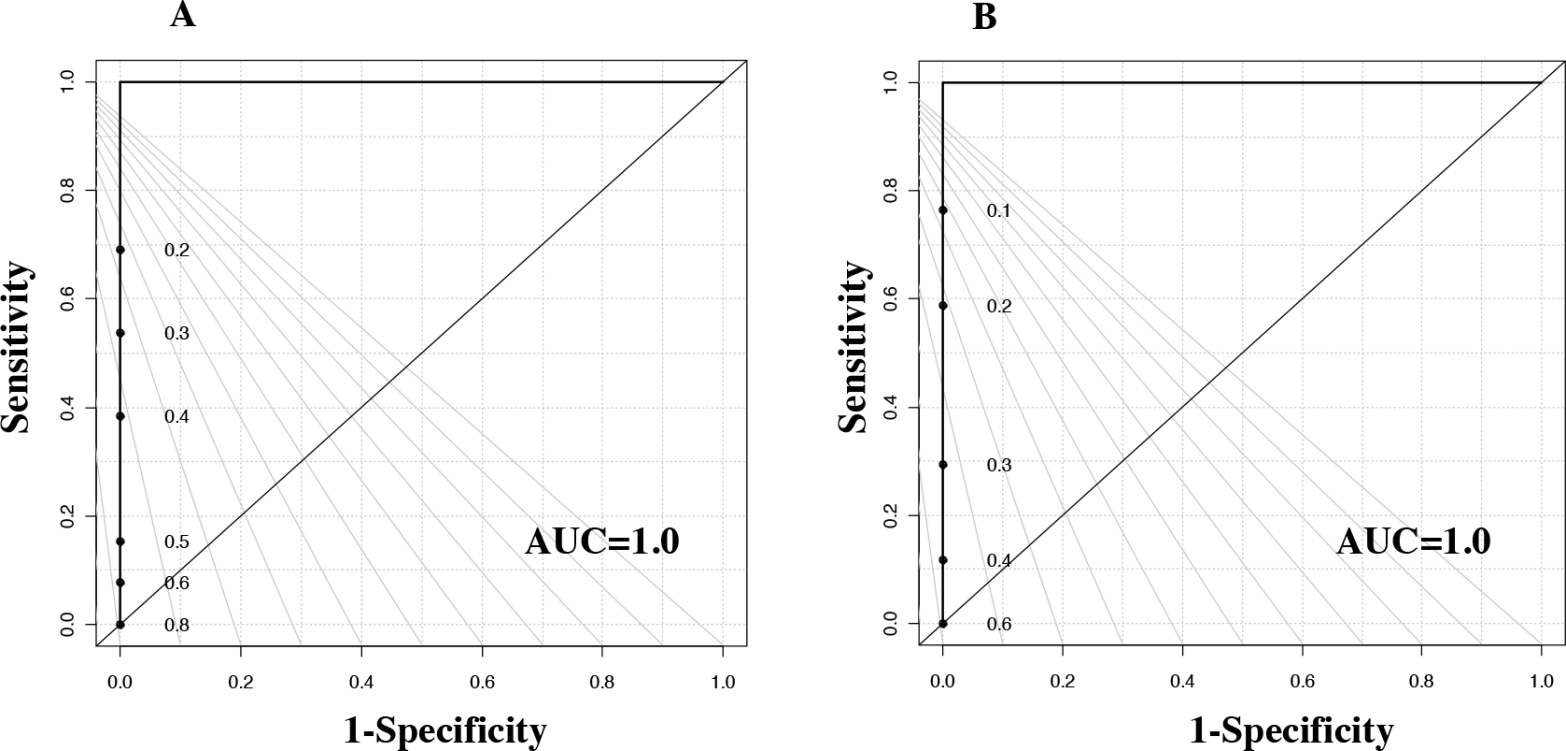
ROC curves for the cross-validated predicted class values obtained using the results of PLS-DA model for African American women with **(A)** TNBC samples and **(B)** LABC samples.

**Figure 6:**
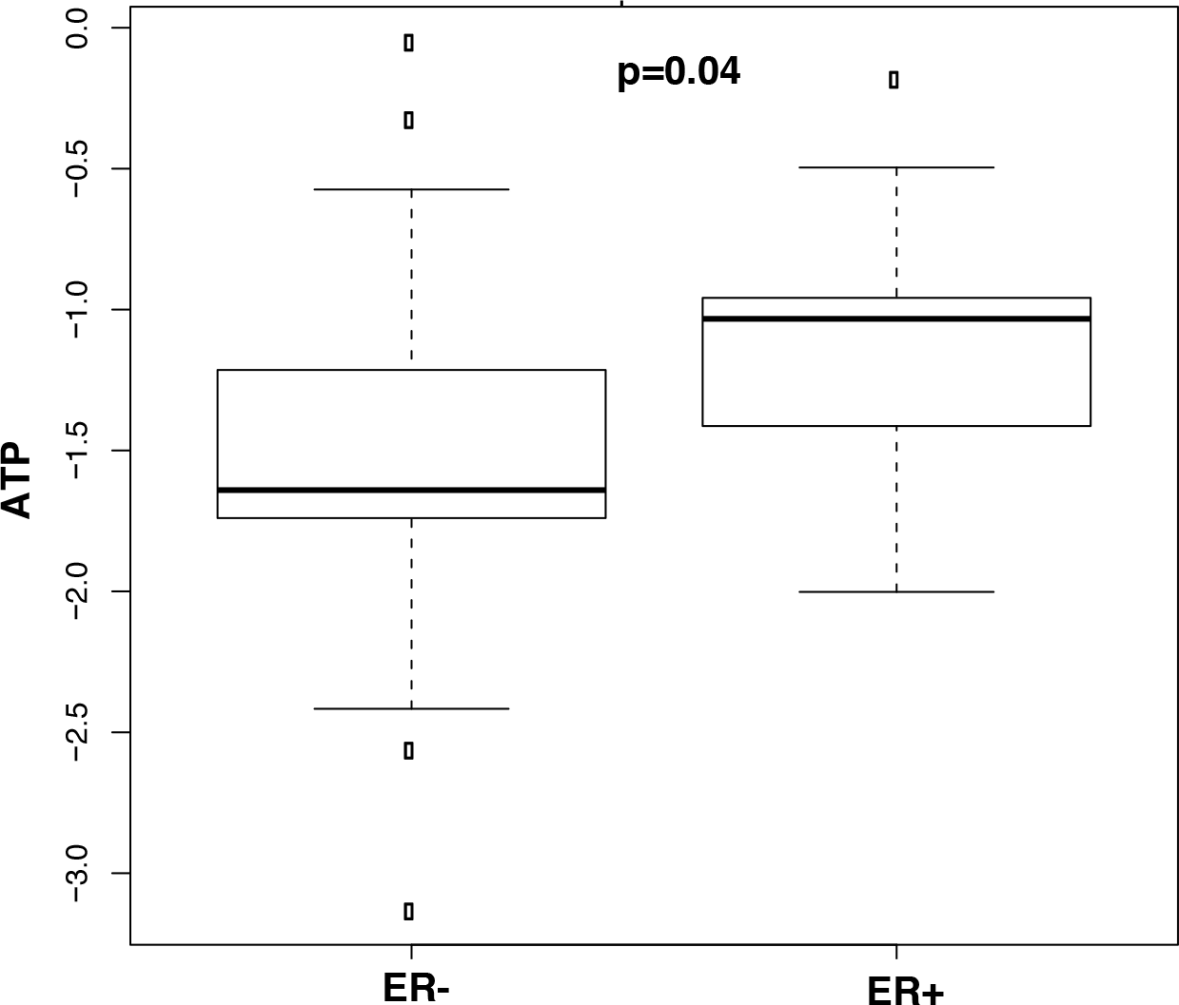
Box-and-whisker plots of metabolites with p-values < 0.05 illustrating discrimination between TNBC vs. LABC in African Americans Horizontal line in the middle portion of the box, mean. Top and bottom boundaries of boxes show the 75th and 25th percentiles, respectively. Upper and lower whiskers show 95th and 5th percentiles, respectively. Open circles show outliers.

For a better understanding of the effect of ER status on tissue metabolites, a PLS-DA model with leave-one-out cross-validation for all 27 metabolites (Supplementary Table 1) was constructed, which showed excellent separation between the tumor and normal adjacent tissues for African-Americans with TNBC vs. their adjacent normal tissues and LABC vs. their adjacent normal tissues, respectively. The performance of the PLS-DA analysis of the above comparisons is displayed using ROC curves (Figure 5A&5B). Relative metabolite levels with p < 0.05 for TNBC/adjacent normal and LABC/adjacent normal are shown Table 4. When comparing the TNBC to the LABC of African-American women, only ATP was found be lower in TNBC and higher in the LABC tissues (Figure 6).

**Table 4:** Metabolites significantly altered (p<0.05) TNBC and LABC tumors *vs*. adjacent normal tissues in African American women

Metabolites	All tumor samples vs. adjacent normal	TNBC samples *vs* adjacent normal	LABC samples *vs* adjacent normal
Alanine	0.005	0.023	-
ATP	0.008	0.035	-
Choline	0.001	0.029	0.019
Glucose	-	0.008	-
Glutamate	0.001	0.024	0.027
Glutamine	0.020	-	-
Glutathione	0.026	0.026	-
Glycine	0.005	0.022	-
Lactate	0.001	0.020	0.02
Lipids	0.019	0.001	-
Lysine, L-Leucine, Arginine	0.008	-	0.041
Methionine	0.002	-	0.023
Myo inositol	0.001	-	0.007
Phenylalanine	0.012	-	-
Phosphocholine	0.005	-	0.009
Taurine	0.004	0.028	0.040
Threonine	0.008	0.022	-
Tyrosine	0.005	0.033	0.043
Valine	0.011	0.036	-
Unsaturated lipids	0.013	0.007	-

### Pathway analysis

**Figure 7:**
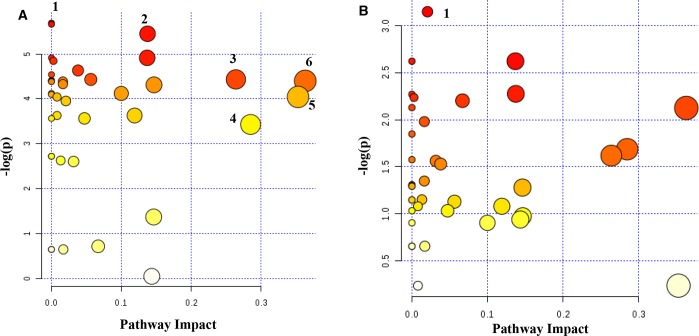
All matched pathways according to p values from pathway enrichment analysis and pathway impact values obtained from pathway topology analysis for **(A)** Metabolites that distinguished tumors and normal adjacent tissues for African Americans. A total of 39 pathways were found to be associated with the metabolites, of which 29 pathways were significant (p<0.05). Glycolysis or gluconeogenesis (1); pyruvate metabolism (2); alanine, aspartate and glutamate metabolism (3); glycine, serine and threonine metabolism (4); glutamine and glutamate metabolism (5); taurine and hypotaurine metabolism (6); **(B)** Metabolites that distinguished tumors and normal adjacent tissues for Caucasians. A total of 39 pathways were found to be associated with the metabolites, of which one pathway, pyrimidine metabolism (1) was significant. (see also Supplementary Table 1)

Pathway enrichment analysis was performed using the metabolite levels to compare the tumor metabolisms of African-American and Caucasian women, and the two groups exhibited striking differences (Figure 7). A total of 39 pathways were enriched in the tumors from African-American women; of these, 29 pathways were found to be significant (p<0.05). In Caucasian women, a total of 39 pathways were enriched; however, only one pathway was found to be significant (p<0.05). The significant pathways associated with the tumor metabolisms of African-American and Caucasian women are listed in Supplementary Table 2.

## DISCUSSION

We have used a metabolomics approach based on HR-MAS NMR spectroscopy and multivariate statistical analyses to determine the altered metabolites levels in breast cancer associated with clinical and demographic parameters. The analysis clearly showed that there were significant differences in the metabolic profiles of tumor and normal adjacent tissues and identified 14 metabolites to be significantly altered due to malignancy. In agreement with our data, the ability of the NMR-based metabolomic approach to distinguish malignant tumor from their adjacent normal tissues in unsupervised and supervised analyses was demonstrated by other studies that examined the metabolomic profile of pancreas, breast, and colon tissues [16–21].

Our results also revealed that the metabolic profiles of breast cancer tissues in postmenopausal (>50 years) women were distinct from those in premenopausal (<50 years) women, which suggest that the metabolic profiles are age dependent, a possible outcome of various aging processes [22]. Few studies have been conducted to compare age-related metabolic effects in women with breast cancer. In one of these studies, it was demonstrated that increased methionine uptake and participation of the transmethylation pathway distinguished TNBC in African-American and Caucasian women younger than 50 years of age [23]. With regard to effect of race on breast cancer metabolism, a number of metabolites were found to discriminate between African-American and Caucasian women in our study. A similar ethnic-based metabolic profile was reported by Stewart et al. [24]. Furthermore, our study showed that LABC and TNBC have different metabolite profiles regardless of race. These tumors could be distinguished from each other with high accuracy (AUC>0.9), which is potentially due to the previously known altered metabolic pathways of these two subtypes [25] [26]. In addition, lipid, unsaturated lipid, and glucose signals were significantly decreased in TNBC, which may be a result of the higher utilization of lipids for membrane biosynthesis and the higher glycolytic activity of these tumors [27]. Interestingly, decreased expression levels of these metabolite was found to have an effect on TNBC patient survival [28].

We then examined our data for the effect of both race and hormonal status. We found that glutathione, choline, glycine, lactate, and glutamine were significantly (p<0.05) higher in the tumor samples compared to the normal tissue samples in African American women. Glutathione, an intracellular antioxidant, plays a significant role in cellular defense [29]; therefore, high levels of glutathione were hypothesized to contribute to the treatment resistance by reducing the effectiveness of drugs intended to damage cancer cells [30] [31]. Higher concentrations of choline and phosphocholine in breast cancer tissues have been reported by several studies [32] [33] [34] [35]. Higher phosphocholine as well as lower glycerophosphocholine and choline levels were shown to be associated with LABC than TNBC. Furthermore, a higher glycerophosphocholine to phosphocholine ratio in TNBC compared to LABC was also observed. Treatment with PI3K pathway inhibitors significantly increased phosphocholine and consequently decreased proliferation in basal-like tumor xenografts but not in luminal-like tumor xenografts [36]. In patients with breast cancer that responded to treatment, choline and phosphocholine levels were significantly lower compared to patients who did respond to treatment. These findings identify a decrease in choline phospholipid metabolism as a potential target of breast cancer therapy [37].

Our pathway analysis visualized the differences in tumor metabolism between TNBC and LABC in African-American and Caucasian women. The same metabolites showed striking differences between the two races. While 29 different pathways showed significant (p<0.05) association with the tumors in African-Americans patients, only one pathway was found to be significant for Caucasians. Pathways associated with energy metabolism—glycolysis, TCA cycle, and amino acid metabolism—were dominant in African-American women, which potentially indicates the aggressiveness of the tumor subtype compared to the tumors of Caucasian women. Our data suggest that TNBC cells in African-American women are more active in glycolysis and ATP metabolism, which was also demonstrated by the lower ATP levels in TNBC compared to LABC. Rapidly proliferating tumor cells undergo metabolic reprograming to meet their unusually high rates of growth and proliferation. Therefore, these cells up-regulate the glycolytic flux to lactate in the presence of oxygen (i.e. the Warburg effect). In this situation, ATP is preferentially generated through aerobic glycolysis instead of oxidative phosphorylation, which leads to the rapid, yet inefficient, production of ATP per unit of glucose consumed [38]. Previous studies also found that TNBC cells exhibited profound metabolic alterations characterized by a decrease in mitochondrial respiration and increased glycolysis [39]. It was documented that rapid ATP consumption (resulting in low levels of ATP) and its degradation product, adenosine, increased breast cancer cell migration. An adenosine receptor antagonist was found to attenuate the ATP stimulation of tumor cell migration and metastases *in vitro* and *in vivo* [40].

However, pathway analysis revealed that only the pyrimidine synthesis pathway is significantly activated in the TNBC of Caucasian women. Pyrimidine nucleotides provide the nucleotide building blocks of RNA and DNA required for cell growth and proliferation. Pyrimidines are synthesized through two routes, either by recycling the nucleotides via salvage pathways or synthesizing de novo from small metabolites through the glutamine-dependent pathway. The activity of the latter is low in normal cells where the need for pyrimidine is largely satisfied by the salvage pathways. In contrast, *de novo* pyrimidine biosynthesis is crucial in proliferating cells in order to meet the increased demand for nucleic acid precursors [41] [42]. It was recently reported that the metabolic reprogramming of pyrimidine synthesis promoted chemotherapy resistance in *in vitro* and *in vivo* TNBC cells. The inhibition of *de novo* pyrimidine synthesis pathway offers a strategy to enhance *in vitro* and *in vivo* sensitivity of TNBC cells to chemotherapy [43]. Furthermore, it was shown that the depletion of glutamine effectively eliminated the ability of chemotherapy to elevate pyrimidine dNTP, suggesting that chemotherapy modulates *de novo* pyrimidine synthesis. Our data showed that glutamine levels were low in the TNBC tissues of Caucasian women compared to the TNBC tissues of African-American women. These findings suggest that glutaminolysis is also upregulated? in these tissues. Thus, TNBC tissues of African-American and Caucasian women exhibit different alterations in cellular metabolism; race as well as hormonal status may play critical roles in breast cancer metabolism. Accordingly, these factors should be investigated in more detail and considered when designing new therapies.

Our study identified that HR-MAS NMR spectroscopy combined with multivariate statistical analysis could be used as a powerful technique to develop the metabolite profiles of breast cancer tissues. Our results revealed numerous statistically significant metabolite changes in tumor tissues compared to the normal adjacent tissue samples of postmenopausal and premenopausal women. Race and hormonal status may affect the metabolite expression of breast cancer. Tyrosine, phenylalanine, and isobutyric acid levels were significantly higher in the LABC of African-American women compared to LABC in Caucasian women. Glutamine was the only metabolite that was found significantly higher in the TNBC of African-American women compared to TNBC in Caucasian women. Importantly, TNBC showed a distinct metabolite profile from that of LABC in African Americans and TNBC in Caucasian women. TNBC in African-American women had reduced ATP level and exhibited profound metabolic alterations characterized by decreased mitochondrial respiration and increased glycolysis. On the other hand TNBC in Caucasian women was associated with increased pyrimidine synthesis. These metabolic alterations in TNBC in both groups of women could potentially be exploited as novel treatment targets.

## MATERIALS AND METHODS

### Chemicals

Deuterium oxide (D_2_O, 99.9% D) was purchased from Cambridge Isotope Laboratories, Inc. (Andover, MA). Trimethylsilylpropionic acid-d4 sodium salt (TSP), sodium azide (NaN_3_), disodium hydrogen phosphate (Na_2_HPO_4_), and monosodium phosphate (NaH_2_PO_4_) were purchased from Sigma-Aldrich (analytical grade, St. Louis, MO).

### Tissues and patient characteristics

A total of 82 human breast tissue samples, invasive grade II-III breast cancer (n=47; 30 African Americans, 17 Caucasian) and normal adjacent (n=35; 18 African Americans, 17 Caucasian) were collected from 47 patients operated on at Indiana University Health, Lafayette, IN; University of Chicago, Chicago, IL; and Indiana Biobank, Indianapolis, IN. Samples were frozen immediately in liquid nitrogen after surgery and then kept at −80 °C until analysis. Purdue University, the University of Chicago, and Indiana University Health Institutional Review Boards approved this work.

### High-resolution magic angle spinning (HR-MAS) 1H NMR

Frozen tissue samples were cut into an appropriate size to fit into HR-MAS sample tubes, and had resulting weights between 11.4 and 22.4 mg. For the field-frequency lock and air removal, 50μl D_2_O was added to each tube. Tubes were then placed into the rotor. 1H NMR experiments for tissue samples were performed on a Bruker Avance-III-800 spectrometer equipped with a HR-MAS probe. NMR data were acquired using the 1D CPMG (Carr-Purcell-Meiboom-Gill) pulse sequence with water presaturation. CPMG experiment parameters included: number of scan=128; number of dummy scans=16; number of time domain points =32K; spectrum width=12,193 Hz; relaxation delay=2s; acquisition time=1.34 s; number of spin-echo loops =400. The acquired spectra were then phased, baseline corrected and referenced to the lipid peak at (δ=0.909 ppm) using Bruker Topspin 3.0 software.

### Data processing and statistical analysis

NMR spectra were binned to 4 K buckets of equal width (0.0034 ppm) to minimize errors due to any fluctuations of chemical shifts arising from pH or ion concentration variations using MestReNova 7.0. The resulting data generated from MestReNova were transferred into Microsoft Excel. Spectral regions between 0 to 9.0 ppm were analyzed after the water region (4.5 to 5.0 ppm) was excluded. The NMR data were normalized to sample weight and the unpaired Student’s t-test was used to identify significant differences in metabolite levels in the tumors and compared with the levels in the normal adjacent tissues. P values ≤ 0.05 were considered as statistically significant. Normalized NMR data were imported into Matlab (R2008a, Mathworks, MA) installed with a PLS-DA toolbox (version 4.1, Eigenvector Research, Inc.) for Partial Least Squares-Discriminant Analysis (PLS-DA). The R statistical package (version 3.0.0) was used for generating box-and-whisker and receiver-operating characteristics (ROC) curves plots. ROC curve analysis using leave-one-out cross-validation was utilized to evaluate the sensitivity and specificity of the PLS-DA model. Pathway enrichment analysis was performed to identify the associated metabolic pathways using MetaboAnalyst 3.0 [44].

## SUPPLEMENTARY MATERIALS FIGURES AND TABLES


