# Supplementary material for: Metabolic profiles of triple-negative and luminal A breast cancer subtypes in African-American identify key metabolic differences
Source: Oncotarget. 2018 Feb 7;9(14):11677–90. doi: 10.18632/oncotarget.24433 (PMC5837744; doi:10.18632/oncotarget.24433)
Supplement: Supplementary file 1 [file oncotarget-09-11677-s001.pdf]

Metabolic profiles of triple-negative and luminal A breast cancer subtypes in African-American identify key metabolic differences

SUPPLEMENTARY MATERIALS

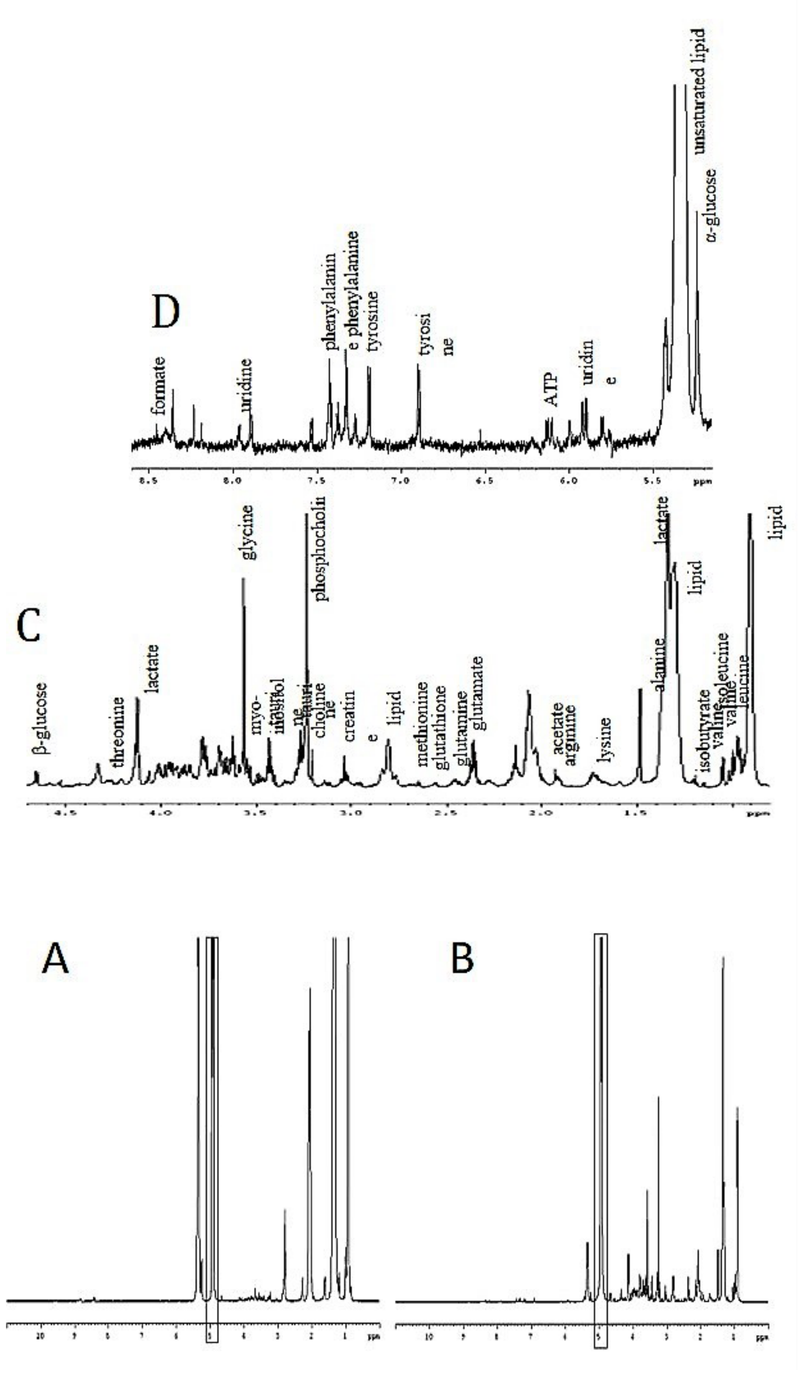

Supplementary Figure 1: HR-MAS ^{1}H NMR spectra of **(A)** a representative adjacent tissue; **(B)** a representative breast cancer tissue with the expanded regions shown in **(C and D)**.

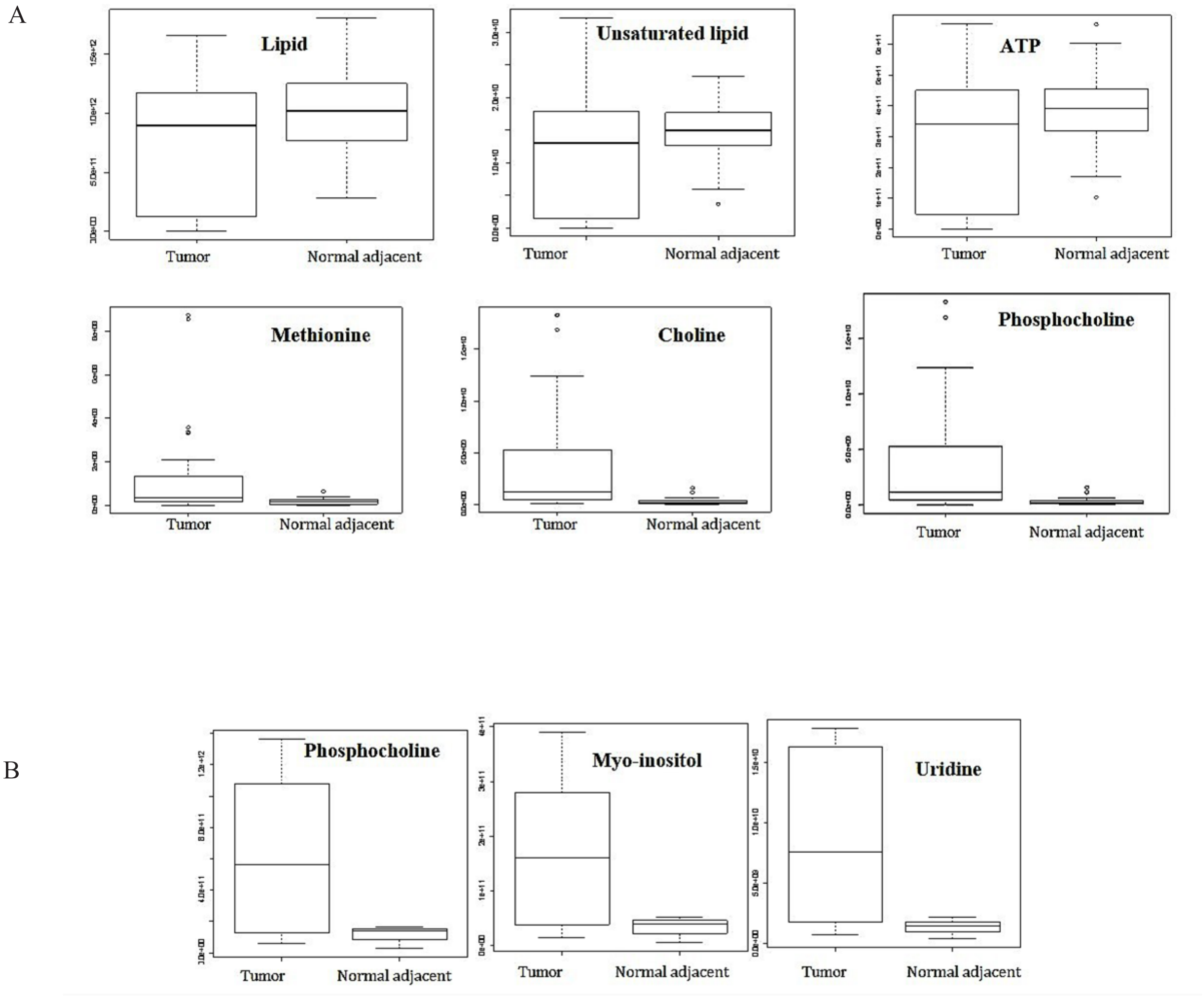

Supplementary Figure 2: **(A)** Box-and-whisker plots of representative metabolites with significantly different ($p< 0.05$) in the tumor compared to normal adjacent tissue for samples from below 50 year-old patients. A horizontal line in the middle portion of the box represents the mean. **(B)** Box-and-whisker plots of metabolites with significantly higher concentration (p-values < 0.05) in tumor compared to normal adjacent tissues for samples from above 50 year-old patients. Top and bottom boundaries of boxes show the 75th and 25th percentiles, respectively. Upper and lower whiskers show 95th and 5th percentiles, respectively. Open circles outside the box indicate outliers.

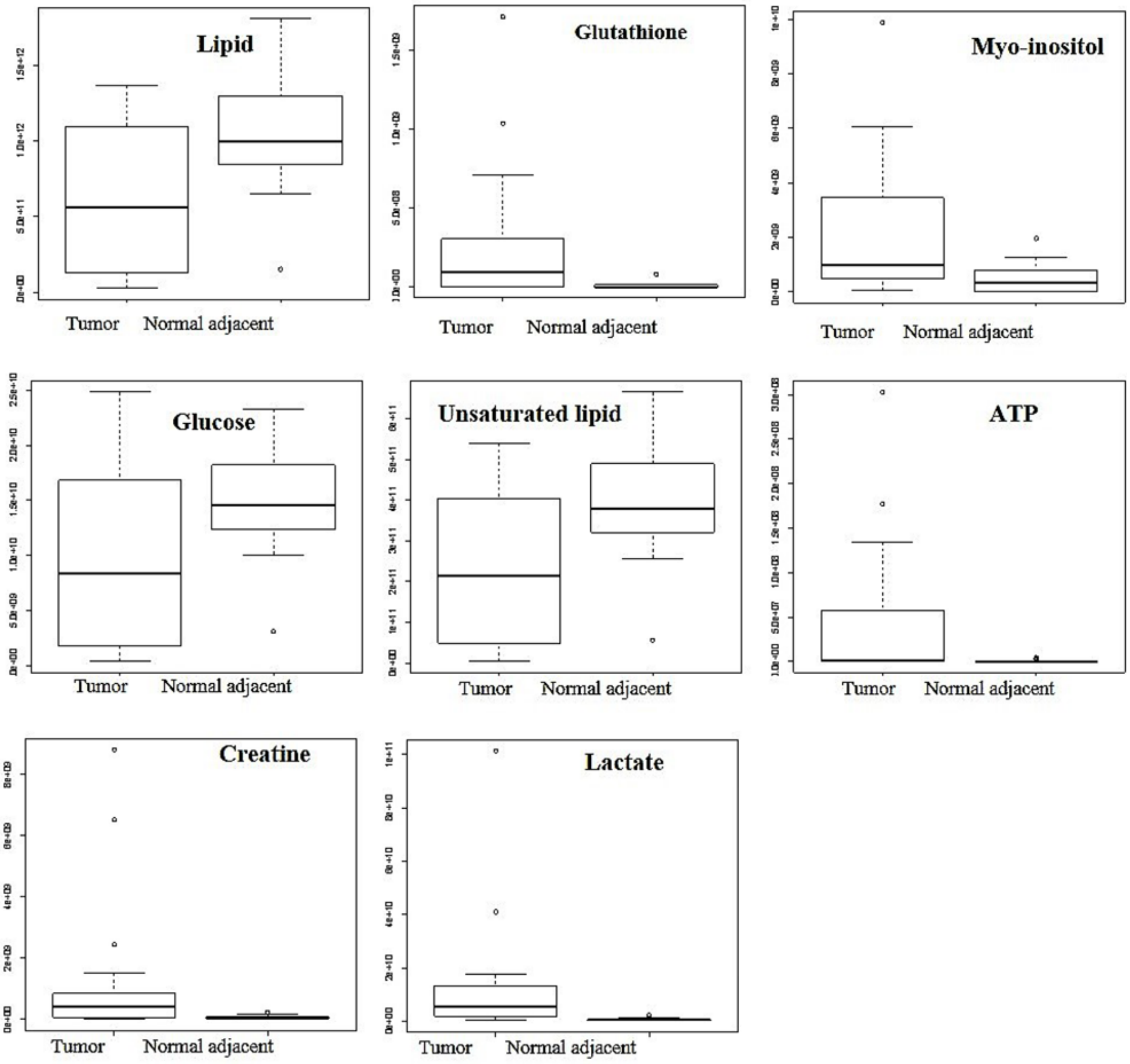

Supplementary Figure 3: Box-and-whisker plots of metabolites with p-values < 0.05 illustrating discrimination between the tumor and normal adjacent tissues for TNBC samples. A horizontal line in the middle portion of the box represents the mean. Top and bottom boundaries of boxes show the 75th and 25th percentiles, respectively. Upper and lower whiskers show 95th and 5th percentiles, respectively. Open circles show outliers.

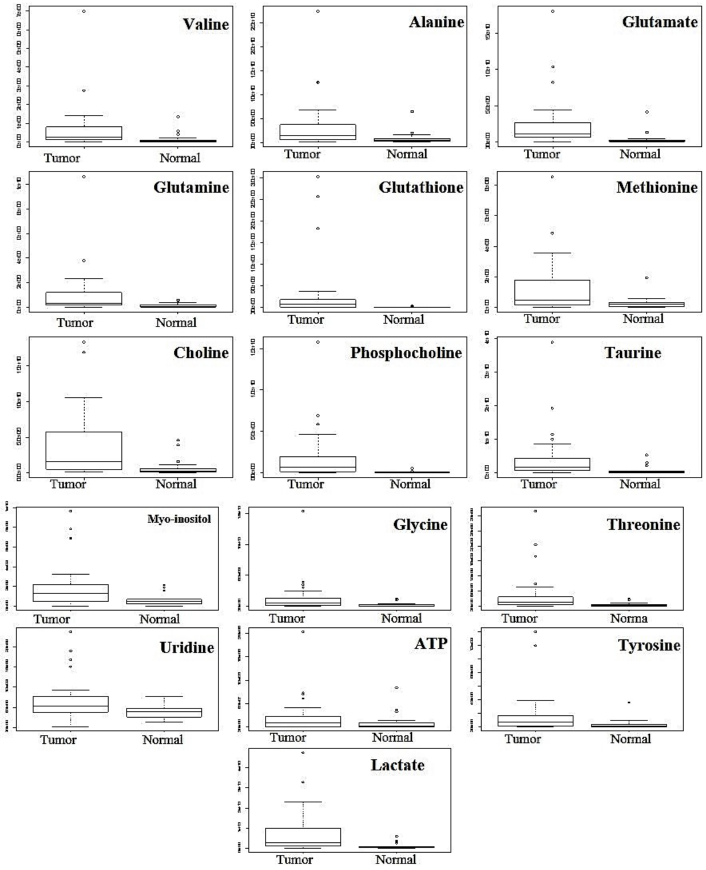

Supplementary Figure 4: Box-and-whisker plots of metabolites with p-values < 0.05 illustrating discrimination between the tumor and normal adjacent tissues for LABC samples. A horizontal line in the middle portion of the box represents the mean. Top and bottom boundaries of boxes show the 75th and 25th percentiles, respectively. Upper and lower whiskers show 95th and 5th percentiles, respectively. Open circles show outliers.

Supplementary Table 1: Quantified metabolites (n=27) in intact breast tumor and adjacent tissue from Caucasian and African American women

Metabolite	Metabolite
Acetate	Leucine
Alanine	Lipid
Arginine	Lysine
ATP	Methionine
Glucose	Myo-inositol
Choline	Phenylalanine
Creatine	Phosphocholine
Formate	Taurine
Glutamate	Threonine
Glutamine	Tyrosine
Glutathione	Unsaturated lipid
Glycine	Uridine
Isobutyrate	Valine
Lactate	

Supplementary Table 2: Comparison of significantly altered metabolic pathways (p<0.05) in African American and Caucasian women with breast cancer

African American		Caucasian	
**Pathway**	**p value**	**Pathway**	**p value**
Glycolysis or Gluconeogenesis	0.0033	Pyrimidine metabolism	0.0428
Propanoate metabolism	0.0034		
Pyruvate metabolism	0.0042		
Inositol phosphate metabolism	0.0073		
Ascorbate and aldarate metabolism	0.0073		
Galactose metabolism	0.0078		
Cysteine and methionine metabolism	0.0097		
Selenoamino acid metabolism	0.0106		
Alanine, aspartate and glutamate metabolism	0.0118		
Aminoacyl-tRNA biosynthesis	0.0118		
Cyanoamino acid metabolism	0.0121		
Thiamine metabolism	0.0122		
Taurine and hypotaurine metabolism	0.0122		
Porphyrin and chlorophyll metabolism	0.0124		
Primary bile acid biosynthesis	0.0124		
Glutathione metabolism	0.0124		
Purine metabolism	0.0132		
Lysine degradation	0.0134		
Lysine biosynthesis	0.0162		
Biotin metabolism	0.0162		
Nitrogen metabolism	0.0166		
Glutamine and glutamate metabolism	0.0175		
Vitamin B6 metabolism	0.0175		
Pyrimidine metabolism	0.0192		
Phenylalanine metabolism	0.0265		
Phenylalanine, tyrosine and tryptophan biosynthesis	0.0265		
Tyrosine metabolism	0.0283		
Ubiquinone and other terpenoid-quinone biosynthesis	0.0283		
Glycine, serine and threonine metabolism	0.0324		
